# A Genetically Encoded Reporter for Real-Time Imaging of Cofilin-Actin Rods in Living Neurons

**DOI:** 10.1371/journal.pone.0083609

**Published:** 2013-12-31

**Authors:** Jianjie Mi, Alisa E. Shaw, Chi W. Pak, Keifer P. Walsh, Laurie S. Minamide, Barbara W. Bernstein, Thomas B. Kuhn, James R. Bamburg

**Affiliations:** 1 Department of Biochemistry and Molecular Biology, and Molecular, Cellular and Integrative Neuroscience Program, Colorado State University, Fort Collins, Colorado, United States of America; 2 Department of Chemistry and Biochemistry, University of Alaska, Fairbanks, Alaska, United States of America; George Mason University, United States of America

## Abstract

Filament bundles (rods) of cofilin and actin (1:1) form in neurites of stressed neurons where they inhibit synaptic function. Live-cell imaging of rod formation is hampered by the fact that overexpression of a chimera of wild type cofilin with a fluorescent protein causes formation of spontaneous and persistent rods, which is exacerbated by the photostress of imaging. The study of rod induction in living cells calls for a rod reporter that does not cause spontaneous rods. From a study in which single cofilin surface residues were mutated, we identified a mutant, cofilinR21Q, which when fused with monomeric Red Fluorescent Protein (mRFP) and expressed several fold above endogenous cofilin, does not induce spontaneous rods even during the photostress of imaging. CofilinR21Q-mRFP only incorporates into rods when they form from endogenous proteins in stressed cells. In neurons, cofilinR21Q-mRFP reports on rods formed from endogenous cofilin and induced by all modes tested thus far. Rods have a half-life of 30–60 min upon removal of the inducer. Vesicle transport in neurites is arrested upon treatments that form rods and recovers as rods disappear. CofilinR21Q-mRFP is a genetically encoded rod reporter that is useful in live cell imaging studies of induced rod formation, including rod dynamics, and kinetics of rod elimination.

## Introduction

In all eukaryotic cells, proteins of the actin-depolymerizing factor (ADF)/cofilin family are key regulators of actin dynamics and actomyosin contractility [1]–[3]. Cofilin is the prominent isoform expressed in mammalian neurons [4]. Neuronal cofilin plays important roles in the synaptic plasticity associated with learning and memory by modulating actin-rich dendritic spine architecture during both ion channel insertion and spine enlargement, two phases of long-term potentiation (LTP) [5], [6].

Under conditions of cellular stress, cofilin forms complexes with actin that can alter cell function [7]. Hippocampal neurons, subjected to energy stress (ATP depletion, excitotoxic glutamate, hypoxia/ischemia) [8], oxidative stress (peroxide, NO) [8], [9], extracellular ATP [10], and soluble forms of the Alzheimer's disease β-amyloid peptides (Aβ) [11], [12], form within their neurites cofilin-actin (1:1) filament bundles called rods [13]. Rod formation requires an intermolecular disulfide bond formed by cofilin oxidation [14]. Cofilin-actin rods can grow to occlude completely the neurite in which they form, thus causing microtubule loss [8] and synaptic dysfunction [15], [16]. Rods are observed in brains from human Alzheimer disease subjects and may even represent a common mechanism compromising synapse function in other neurodegenerative diseases.

Because rods form from minutes to hours in stressed neurons, they may be an early event in the neurodegenerative cascade and an ideal target for therapeutic intervention in treating many different neurodegenerative disorders [17]. However, when fluorescently tagged wild type (wt) cofilin is used to image rods in living cells, its overexpression in the absence of other stress induces rod formation, which is exacerbated by the photostress of microscopy [16], [18]. Rods formed by overexpression and photostress confound the interpretation of studies designed to monitor induced rods and their effects on cell biological processes, rod dynamics and rod reversibility.

Here we report on studies in which various promoters were used to reduce expression of wt cofilin fluorescent protein chimeras to determine if decreased expression alone would render wt cofilin chimeras an acceptable genetically encoded rod “marker”. We also characterized surface residue mutants of cofilin to identify mutants that 1) do not form rods when overexpressed in unstressed cells and 2) are incorporated into rods formed by endogenous cofilin in stressed cells. We then used one of these mutants, cofilinR21Q fused to mRFP, to study rod dynamics and effects of rod formation and reversal on vesicle transport in neurons.

## Materials and Methods

### Materials

All unspecified chemicals were reagent grade and were obtained from Sigma-Aldrich. 2-Amino-3-(3-hydroxy-5-methyl-isoxazol-4-yl)propionic acid (AMPA) (made as 25 μM stock in DMSO) was from Ascent Scientific and 6,7-dinitroquinoxaline-2,3-dione (DNQX) (made as 50 μM stock in water) was from Tocris Bioscience. Cell culture reagents were from Life Technologies and fetal bovine serum (FBS) from Hyclone Labs. Wild type cofilin, cofilinR21Q and cofilinK22Q were purified from a bacterial pET expression system using chromatography on DEAE cellulose (Whatman) and Green A dye matrix resin (Millipore) as described for chick ADF [19], but with adjustments in pH for the Green A resin to optimize binding of the more negatively charged cofilin mutants. Actin was purified from chicken muscle acetone powder and gel filtered [20]. Analysis of purity of actin and cofilin (Figure S3 in File S1) was performed by methods previously reported [21] and was greater than 99%.

### Ethics Information

All rats were handled according to National Research Council's Guidelines to Care and Use of Laboratory Animals as approved by the Colorado State University Institutional Animal Care and Use Committee (approved protocol #11-3951A).

### Cell Culture

Pig kidney LLC-PK_A4.8_ cells [22], SAOS2 osteosarcoma cells [23], HeLa cells [24], and N2a neuroblastoma cells [25] were obtained from referenced source and cultured as described therein. HEK 293 cells were grown in tissue culture dishes in high glucose Dulbecco's Modified Eagle Medium (HGDMEM) containing 10% fetal bovine serum (FBS). E18 rat hippocampal neurons were obtained by dissection from timed-pregnant Sprague-Dawley dams [26] and were dissociated and frozen for storage in liquid nitrogen [27]. Aliquots of frozen neurons were rapidly thawed at 37°C and plated onto poly-D-lysine coated 170 μm thick German glass coverslips (22×22 mm; Carolina Biological Supply) fixed to the bottom of drilled out 35 mm petri dishes or T25 tissue cultural flasks with aquarium sealant and cured for 24 hours before plating neurons. Cells were grown in Neurobasal medium with B27 supplement (200 μl/10ml) and Glutamax I (25 μl/10ml) at a density of 2×10^4^ cells/120 mm^2^ [28]. For imaging of APP-YFP transport, neurons were switched to Hibernate-A medium (BrainBits) supplemented with the same components as in the complete Neurobasal medium but incubated without CO_2_.

### Adenovirus Production

Adenoviruses were made, amplified, and titered using the AdEasy system [29] as modified [30]. To make the pShuttle-MCP (mouse cofilin promoter), a 1255 bp region of the mouse cofilin 1 gene in BAC RP-23-457M2 (NIH BAC Resource Network) just upstream of the start codon was amplified by PCR using the following primers containing a KpnI site: 5′ TTTCTAGATGGTACCGCTTCGGCCTCCACCTGG, 3′ TCTTCTAGAGGTACCGGGAGACAGAAAGAGCAACTG. KpnI-cut MCP DNA was then ligated into the KpnI site of pShuttle. A 710 bp polyadenylation sequence from the 3′UTR of the mouse cofilin-1 gene was amplified from the BAC template using PCR primers that contained a BglII site using the following primers: 5′ TTCAAGATCTGCCGTCATTTCCCTGGAGG, 3′ TTCAAGATCTGAGCCCAACTGCCCTGCC. This polyadenylation sequence was also cloned into the BglII site of pShuttle to generate pShuttle-MCP. pShuttle vector with a neuronal specific enolase (NSE) promoter in place of the CMV promoter was made by PCR amplification of a 1.1 kb portion of the rat NSE gene template [31] using a forward primer containing AseI and AflII and a reverse primer containing BglII and PvuI sites. Primer sequences are as follows: GGGATTAATCTTAAGGGGACAGTAAAGGTGATGGC, 3′ GGGAGATCTCGATCGGAGGACTGCAGACTCAGCC. The PCR product was digested with AseI and BglII and cloned into the AseI and BamHI cut pmRFP-N1 in place of the CMV promoter. The mRFP was removed from this plasmid with PvuI and XbaI and replaced with the PmeI-excised multi-cloning site of pcDNA3.1. The resulting NSE promoter/multicloning site/polyadenylation signal sequence cassette was excised by AflI partial digestion, the DNA fragment agarose gel purified, and then ligated (blunt end) into pShuttle cut with KpnI and SalI. Plasmids with correct orientation were identified by test digestion. The human cofilin-mRFP cDNA in pmRFP-C1 vector [18], [32] was mutated by PCR-based site-directed mutagenesis (Stratagene QuikChange Mutagenesis Kit) to generate the R21Q using the following primers: 5′ CGACATGAAGGTGCAGAAGAGCTCAACCCCAGAGG, 3′ CCTCTGGGfGTTGAGCTCTTCTGCACCTTCATGTCG. The cofilin region was sequenced to ensure correct modification and the cDNAs encoding cofilin wild type and R21Q mutant-mRFP chimeric sequences were cloned into the multicloning sites of pShuttle-CMV, pShuttle-MCP and pShuttle-NSE. Amyloid precursor protein-YFP, a generous gift from Lawrence Goldstein, was subcloned into pShuttle CMV. For shRNA targeting cofilin in LLC-PK_A4.8_ cells, adenovirus made for use in mouse cofilin silencing was used [4]. The human cofilin target sequence of AAGTCTTCAACGCCAGAGGAG was used for making the human shRNA construct in the same manner. For adenovirus production, vectors were linearized, electroporated into BJ5183 *E. coli* containing the AdEasy virus DNA, bacteria containing homologous recombinants within the AdEasy DNA were selected, viral DNAs containing the different promoter driven cofilin constructs were isolated, linearized and transfected into HEK293 cells, and the produced virus amplified twice more and titered [30].

### Adenoviral Infection

For determination of promoter strength, SAOS2 cells, HeLa cells, and N2a neuroblastoma cells were infected with adenoviruses at 25–50 multiplicity of infection (m.o.i) and cell extracts prepared between 48 and 72 h post-infection. Promoter expression strengths were determined by quantifying the relative intensity of the cofilin-mRFP band (47 kDa) to that of endogenous cofilin (19 kD). In N2a cells in which all promoters are active the ratio was ∼5× for CMV, ∼2× for the mouse cofilin promoter, and ∼1.5× for the NSE promoter (Figure S1 in File S1).

E18 rat hippocampal neuronal cultures were maintained in a 5% CO_2_ incubator at 37°C for 3 days before medium change and infection at 100–300 m.o.i as described previously [4]. Cultures were incubated overnight before half of medium was replaced. For most experiments other treatments occurred on day 5 *in vitro* with imaging at that time or one day later. APP-YFP trafficking studies were done on day 6 *in vitro*.

### Western Blotting

Extracts from cell lines infected with adenoviruses for cofilin-mRFP expression were made as previously described [33] and subjected to sodium dodecyl sulfate-polyacrylamide gel electrophoresis and immunoblotting using an affinity purified mammalian ADF/cofilin pan antibody (rabbit 1439) [34] and Dy-Light conjugated secondary antibodies (ThermoFischer). Blots were quantified by scanning using an Odyssey IR laser scanner (Li-Cor Biosciences) and quantified using TotaLab software (Non-Linear Dynamics).

### Amyloid Beta Peptide Preparation

Culture medium of 7PA2 Chinese hamster ovary cells expressing a human amyloid precursor protein with AD mutations [35] was concentrated 10× and gel filtered on Superose 75 resin in a volatile ammonium acetate buffer [36]. The two fractions containing SDS-stable Aβ dimer/trimer (Aβd/t) were detected by immunoblotting [12], combined and freeze dried.

### Cell Treatments

Neurons were treated with Aβd/t by reconstituting freeze dried aliquots in Neurobasal medium at the final concentration used in neuronal cell culture (equal to its concentration in the 7PA2 medium), estimated by immunoblotting against synthetic human Aβ_1-42_ to be approximately 250 pM [12]. Cells with a medium change served as controls. ATP depletion was performed as previously described [8]. Cell transfection was performed with Lipofectamine (Life Technologies) according to the manufacturer's protocol.

### Fixation and Immunolabelling

Neurons were fixed for 45 min, at room temperature in 4% formaldehyde in phosphate buffered saline (PBS), permeabilized with methanol (−20°C) for 3 min and blocked in 2% goat serum/1% bovine serum albumin in TBS (10 mM Tris pH 8.0, 150 mM NaCl) before immunolabelling. Affinity purified rabbit 1439 IgG (2 μg/ml in blocking buffer) was applied to the cells for 2 h at room temperature or overnight at 4°C. After rinsing 5× with TBS, secondary antibody, either Alexa 488 or Alexa 647 conjugated goat anti-rabbit (Molecular Probes), was applied at 1:400 dilution for 1 h at room temperature. After washing in TBS, coverslips were applied to slides with ProLong Gold Antifade (Molecular Probes).

### Fluorescence Imaging

Routine screening and rod quantification was performed on images captured on an epifluorescence Nikon diaphot microscope with 40× or 60× oil objectives equipped with a Photometrics CoolSnap ES camera controlled by Metamorph software. Live cell imaging was performed on a heated automated stage of either: (1) an Olympus IX81 inverted microscope equipped with a Yokagawa spinning disk head, 4 lasers, a 60× oil 1.42NA DIC objective, and a Cascade II EMCCD, integrated by Intelligent Imaging Systems (3I) and operated by Slidebook software, or (2) a Nikon Eclipse Total Internal Reflection Fluorescence (TIRF) inverted microscope with perfect focus, 100× oil 1.47 NA TIRF objective or a 40× 0.75 NA air objective, motorized stage with stage incubation system and CO_2_ control, an Andor iXon3 EMCCD camera, and operated by Nikon elements software for image acquisition. Unless otherwise specified, time lapse images were collected every 30 s for about 2 h. Exposure times for fixed cells were adjusted for each fluor to avoid saturation, but in studies on live cells rods often developed that reached saturation. Captured images were inverted to enhance the appearance of rods for subsequent analysis but rod figures are pseudocolored to reflect the labels used.

### F-Actin Binding Assay

Various concentrations of cofilin (0, 2.5, 5, 10 and 20 μM) were incubated with 5 μM F-actin and samples were sedimented at 436,000 g for 30 min in a TLA-100 rotor at 20°C in a Beckman TL-100 centrifuge. Aliquots of the sample before centrifugation and the supernatant and suspended pellet after centrifugation were boiled in SDS sample preparation buffer, proteins separated by electrophoresis on 15% isocratic SDS-polyacrylamide gels, and proteins stained and quantified as previously described [21].

### Analysis and Statistics

For quantification of cells containing rod structures, neurons with and without rods were counted from randomly selected fields on each coverslip, and the percent rod index is the percentage of total cells that contained one or more rods. To ascertain the relative abundance of rods in a region of the coverslip for cells with uniform plating density, or as a number of rods per neuron (number rod index), the number of rods per field from at least 20 random fields (normalized to cell number if neuronal distribution was uneven) was recorded. Unless otherwise stated, all experiments were repeated a minimum of three times using independently prepared cell cultures. Levels of significance compared to the control (single condition variable) were calculated using the student “T-test” and are reported as significant if p<0.05.

## Results and Discussion

### Even modest overexpression of wild type cofilin results in spontaneous rod formation

**Figure 1:**
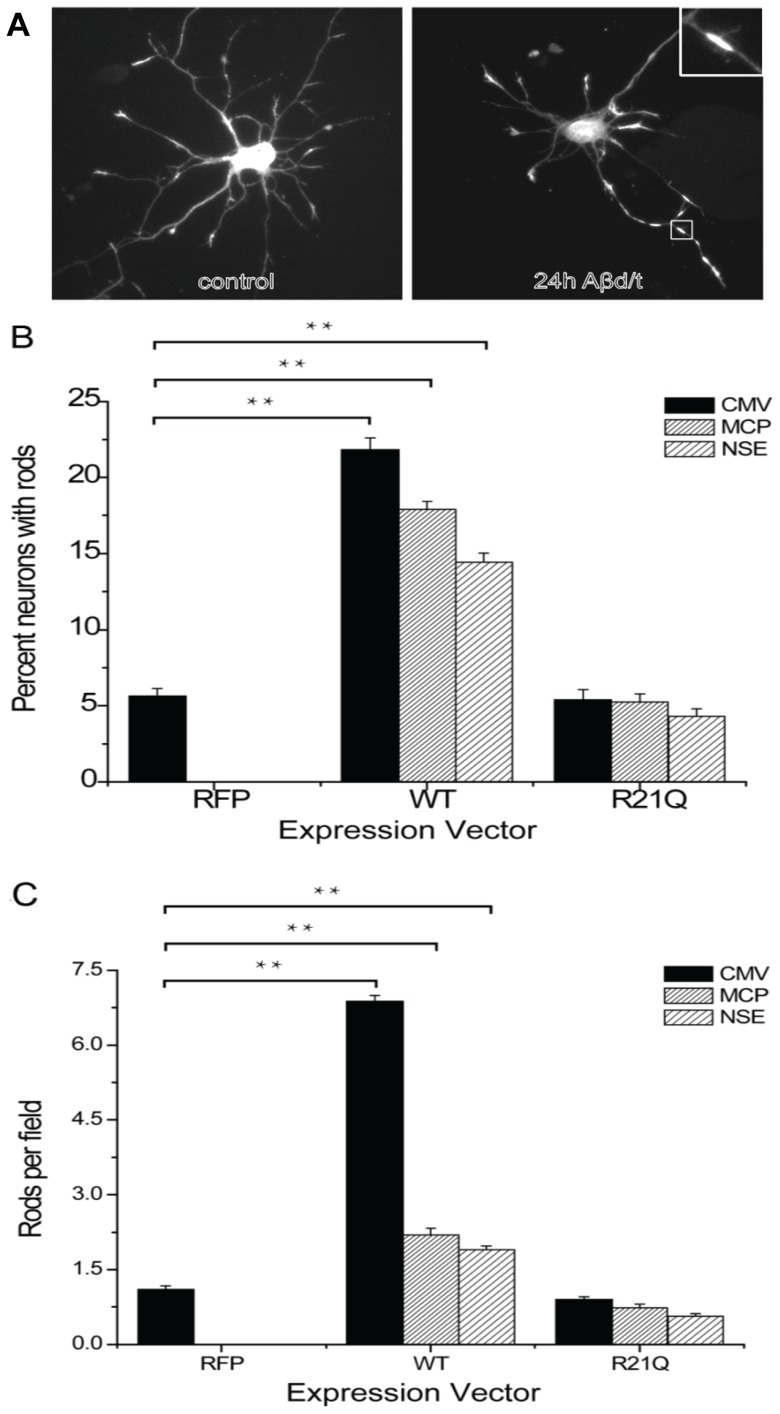
Quantification of rods in cultured neurons expressing either WT or R21Q cofilin-mRFP from promoters of different strengths. (A) Cofilin-immunolabled hippocampal neurons (5 days in vitro) untreated (control) or treated with Aβd/t for 24 h, which induced rods in some neurites. Inset: higher magnification of the rod boxed in the figure (B) The percentage of neurons (percent rod index) that form spontaneous rods is significantly above control levels and proportional to the level of expression of wild type cofilin-mRFP. Controls for this and all other experiments for which proteins are expressed via adenovirus infection are neurons infected with adenovirus expressing mRFP alone, which in all cases gave percent and number rod indexes identical to untreated cultures (not shown). The percent rod index remains at control levels and is independent of the level of expression of cofilinR21Q-mRFP. (C) The number of rods (rod number index) forming per neuron (or field for equal density cell cultures) is significantly above background and proportional to the level of expression of wild type cofilin-mRFP. The number rod index is not increased above control level for neurons expressing cofilinR21Q-mRFP. (**Significant at p<0.005, compared to the CMV-RFP control group).

Cofilin-actin rods formed from endogenous proteins are induced in neurons under stress [8], [11], [12], [14] and may be observed by fixation and immunolabelling for cofilin as shown in Figure 1A. To identify a genetically encoded marker for visualizing rods in live cells, we first examined the possibility of expressing monomeric Red Fluorescent Protein (mRFP) chimeras of wild type cofilin. Typically, overexpression of tagged wild type (wt) cofilin has been driven by high-expression promoters, such as CMV, and resulted in overexpression levels 3–5 fold higher than normal. Thus, we wondered if spontaneous rods could be prevented by curbing overexpression. To determine if low to moderate expression levels of tagged wt cofilin could be used to follow induced rod formation without increasing spontaneous rods, we used adenoviruses containing promoters of different strength to drive different levels of expression of chimeric cofilin-wt-mRFP. Levels of expression relative to endogenous cofilin were quantified by Western blotting in multiple cell lines in which nearly 100% infection could be achieved. Results from one cell line (N2a cells) in which all promoters are active are shown in Figure S1 in File S1. Using the three promoters, we then determined if simply limiting the expression levels of cofilin-wt-mRFP would reduce numbers of neurons in which spontaneous rods form and/or reduce the numbers of rods in these neurons. The percent rod index is the percent of neurons that contain at least one rod (counted from a minimum of 100 neurons per culture and three separate cultures per experiment). The number rod index is the number of rods scored per field in cultures of equal density (or as rods per field divided by cell soma per field if culture density was quite variable) determined from at least 20 random fields per culture and three cultures per experiment. As a control for adenovirus infection, stress induced from mRFP expression alone, and photostress during imaging, we infected cells with adenovirus expressing only mRFP behind a CMV promoter. As shown in Figure 1B, about 5% of uninfected cells form spontaneous rods (with a number rod index shown in Figure 1C of about 1 rod per field); neither adenovirus infection nor mRFP expression affect spontaneous rod formation. However, expression of cofilin-wt-mRFP increases significantly the percentage of neurons with rods and also increases the number rod index, showing that even moderate expression of wt cofilin results in a significant increase in spontaneous rods (Figure 1B, C).

### Photostress of imaging also contributes to an increase in spontaneous rods

Even when rods have not spontaneously formed following overexpression of cofilin-wt-mRFP, cells within the light path during imaging are more likely to form rods than those outside the light path [18]. To determine if the numbers of rods induced by photostress also depend upon the levels of expressed protein, we followed rod formation over 2 h at 30 s intervals in cultured E18 hippocampal neurons infected with adenovirus on day 3 and imaged on day 5. The number of newly formed rods was quantified from several different cultures and the averages are shown in Figure S2 in File S1. As found for spontaneous rods, photostress induces rods in proportion to levels of cofilin-wt-mRFP expressed in cells.

### Identification of cofilin mutants whose overexpression does not induce spontaneous rods

**Figure 2:**
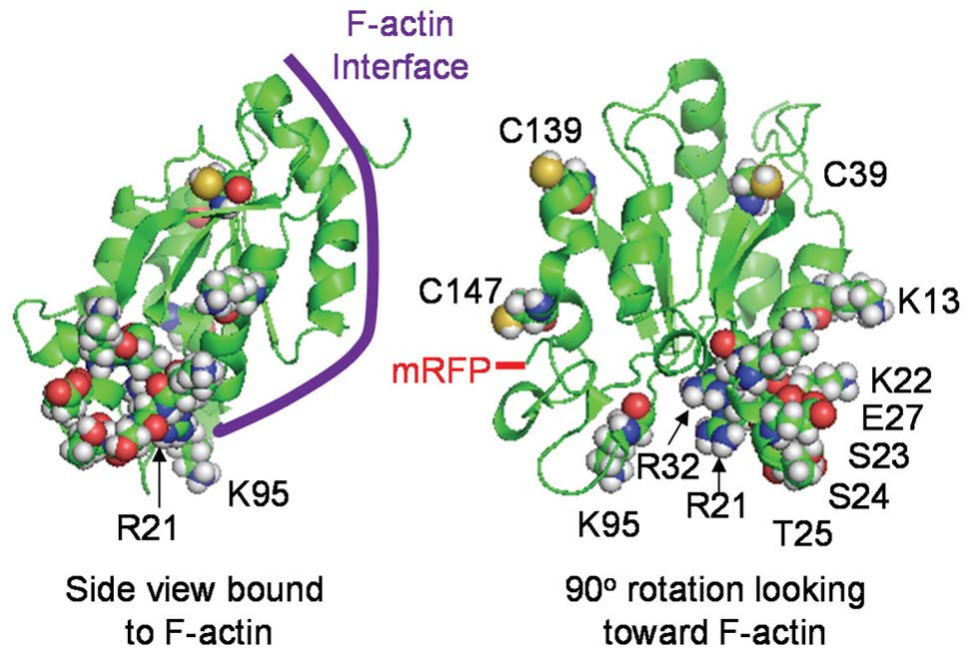
Cofilin structure and position of residues for which site directed mutants were made. Side view of cofilin model created in PyMOL showing approximate F-actin binding interface for both upper and lower subunit contacts, and a rotated view showing the position of the residues for which mutants were made, as well as the C-terminal residue through which the mRFP is connected.

**Figure 3:**
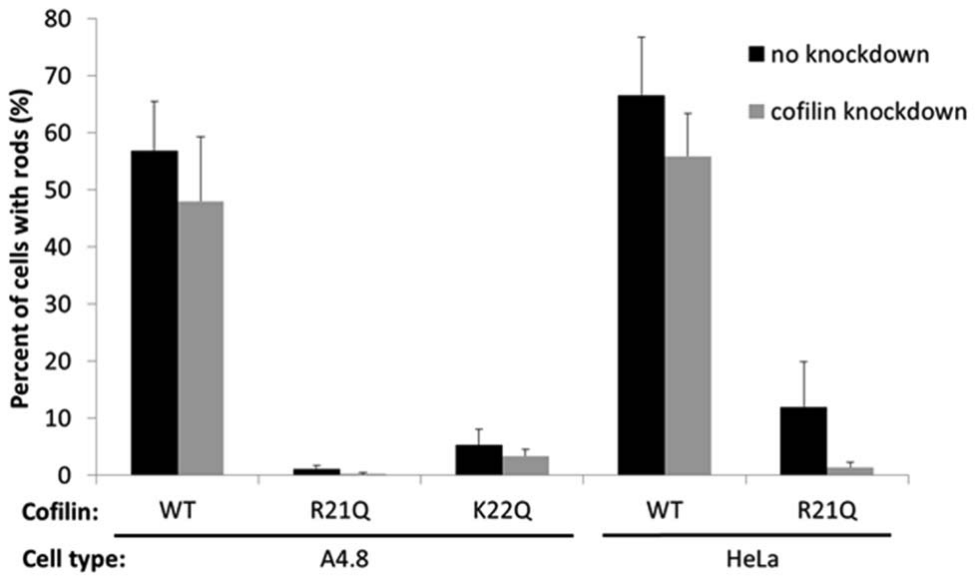
Rod formation in cells overexpressing either cofilinR21Q-mRFP or cofilinK22Q-mRFP is dramatically reduced compared to expression of cofilin-wt-mRFP. Pig kidney LLC-PK_A4.8_ cells and HeLa cells were infected with adenovirus for expression of a cofilin shRNA (grey bars) or a control adenovirus (black bars), then 3 days later the cells were transfected or infected with plasmids or adenovirus for expressing cofilin-mRFP chimeras. Two days after this, the cells were ATP-depleted. The percentage of mRFP-positive cells forming rods is shown. Uninfected LLC-PK_A4.8_ or HeLa cells that are ATP-depleted, fixed and immunolabeled for cofilin contain no rods [22]. Based upon quantification of cofilin on Western blots of cell extracts (data not shown), the silencing in both cell types is >95%.

To circumvent the problems associated with spontaneous rod formation in studying induced rods, we sought to identify a cofilin mutant that would not form spontaneous rods when overexpressed while still serving as reporter for induced rods. Although there is no high resolution molecular structure of cofilin bound to F-actin, several models exist derived from low resolution methods and molecular dynamics simulations [37], [38]. Based on these current models, we performed candidate-based site-directed mutagenesis of several residues on the cofilin surface thought not to be in direct contact with F-actin (Figure 2). We chose to mutate some basic residues (K to Q to maintain similar side chain sizes), some cysteine residues (C to A) because of the role of oxidation in rod formation [14], and then other residues surrounding the major site which we eventually identified as a region with rod-reporter potential. We expressed mRFP chimeras of wt and 13 mutants (K13Q, R21Q, K22Q, SS23,24AA, T25A, T25G, E27A, E27G, KR31,32TL, K95Q, C39A, C139A, and C147A) by transfection of plasmids into LLC-PK_A4.8_ cells. The LLC-PK_A4.8_ cells express endogenous ADF/cofilin at levels too low to form rods when ATP depleted. However, ATP depleted LLC-PK_A4.8_ cells expressing cofilin-wt-mRFP form abundant rods. Conversely, ATP depletion of cells expressing either cofilinR21Q-mRFP or cofilinK22Q-mRFP show greatly decreased rod formation (Figure 3), suggesting that they do not to initiate rod formation on their own. The cDNAs used for making all cofilin-mRFP expression plasmids contain silent mutations making them resistant to shRNA in HeLa cells. Also when expressed in HeLa cells silenced for endogenous cofilin, these two mutants do not initiate rod formation (Figure 3).

We previously showed that the R21Q mutant of cofilin has reduced F-actin binding affinity [14], perhaps contributing to its reduced ability to form spontaneous rods. We wanted to determine if the decreased ability of cofilin K22Q to form spontaneous rods derived from the same mechanism. To compare quantitatively the interactions between cofilin wt, R21Q and K22Q with F-actin, we bacterially expressed and purified them, and tested their F-actin binding in a steady state pelleting assay. Surprisingly, the affinity of cofilin K22Q for F-actin was considerably greater than R21Q, although still lower than wt cofilin (Figure S3 in File S1). Both the R21 and K22 residues are near the interface for the lower subunit along F-actin (Figure 2), but it is unclear based upon current models why the R21Q should have such a reduced actin binding. These two residues make up part of a bipartite nuclear co-localization motif and hence are thought to be surface exposed [39]. There is evidence that cofilin undergoes some conformational changes during F-actin binding [40], but unfortunately the region under discussion is one for which only a low resolution electron microscopy map is available [37], and no changes in cofilin conformation in this region have been determined. Nevertheless, we chose to utilize the R21Q over the K22Q mutant because overexpression of a more fully functional cofilin would likely disrupt many aspects of cell behavior and a weaker F-actin binding mutant should be less disruptive.

### CofilinR21Q-mRFP functions as a genetically encoded rod reporter

We made adenoviruses for expressing cofilinR21Q-mRFP behind the same series of promoters we used for expressing cofilin-wt-mRFP and compared both the percent rod index and the number rod index in cultured E18 rat hippocampal neurons (Figure 1). Unlike neurons expressing cofilin-wt-mRFP, in which spontaneous rods form proportional to the level of expression, the percent of neurons forming rods and the number rod index for all vectors expressing the R21Q mutant are statistically identical to rod formation in the control neurons (either uninfected or expressing mRFP alone). Thus, overexpression of cofilinR21Q-mRFP, even behind the strong CMV promoter, does not induce spontaneous rods. In addition, no new rods form in cofilinR21Q-mRFP-expressing cells during an equivalent period of photostress, which induced new rods in cells expressing the cofilin-wt-mRFP (Figure S2 in File S1).

**Figure 4:**
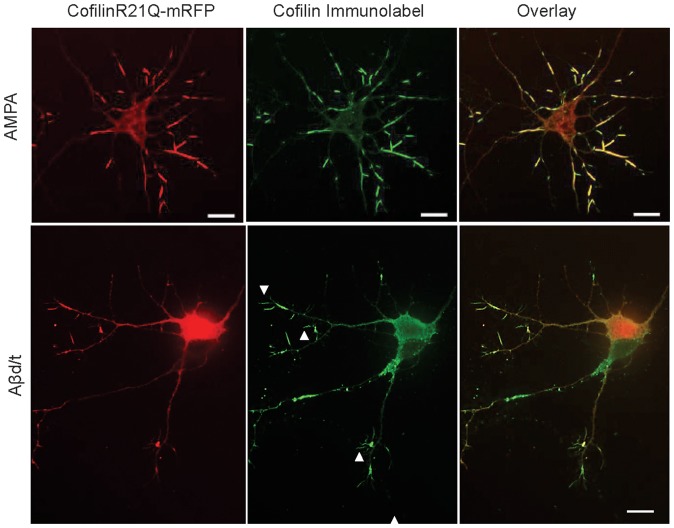
CofilinR21Q-mRFP incorporates into virtually all AMPA-induced rods, but only into half of rods in Aβd/t-treated neuronal cultures. CofilinR21Q-mRFP fluorescence image, immunolabeled (Alexa 488) image, and overlay in neurons treated 30 min with 150 μM AMPA showing virtually all rods (immunolabel) have incorporated cofilinR21Q-mRFP. Similar results (not shown) were obtained for ATP-depleted neurons. CofilinR21Q-mRFP fluorescence image, immunolabeled image (Alexa 647 but colorized green), and overlay in neurons treated 24 h with Aβd/t. Immunolabeled rods that incorporated cofilinR21Q-mRFP were quantified from many different cultures and co-labeled rods accounted for 48±4% (std. deviation) of the total rods. Immunolabeled rods that do not contain mRFP are shown by arrowheads. Scale bars  = 10 μm.

To determine if the cofilinR21Q-mRFP could be used as an *in vivo* reporter for rod formation, we infected neurons with the various adenoviruses (including the mRFP control) on day 3 in culture. On day 5 we either treated neurons with the rod-inducing glutamate analog AMPA [14] or ATP-depletion medium [8], both of which rapidly (20–30 min) induce rods in >80% of neurons. Neurons were fixed and stained for rods. Greater than 80% of treated neurons contain cofilinR21Q-mRFP-labelled rods, and over 98% of rods identified by immunolabelling contain cofilinR21Q-mRFP (Figure 4), indicating that cofilinR21Q-mRFP is an effective reporter for rods induced by AMPA or ATP-depletion.

**Figure 5:**
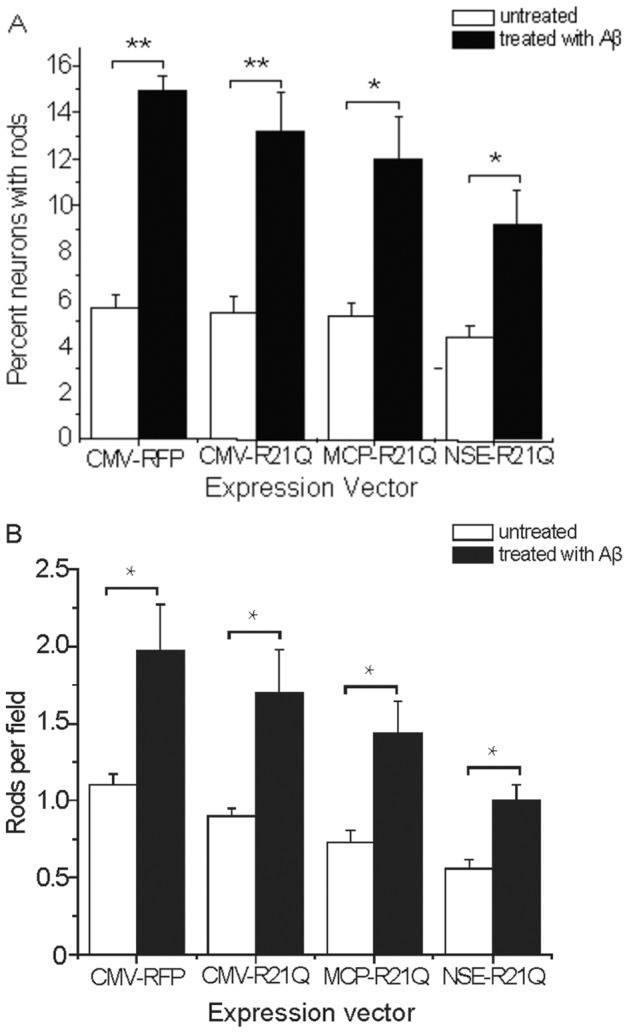
CofilinR21Q-mRFP reports on rod formation in hippocampal neurons in response to Aβd/t treatment. (A) The fraction of cofilinR21Q-mRFP expressing neurons that formed rods after treatment with Aβd/t is 2 to 3 fold higher than for untreated neurons, regardless of which promoter drives expression, although more rods are detected when cofilin-R21Q-mRFP expression is greatest. (B) The number of rods per field in Aβd/t treated neurons expressing cofilinR21Q-mRFP is about 2 fold higher than the corresponding non-Aβd/t treated controls regardless of which promoter drives expression. (*Significant at p<0.05, **Significant at p<0.005, compared to their appropriate non-Aβd/t-treated control group).

We also treated infected neurons with a dimer/trimer fraction of cell secreted Aβ (Aβd/t) on day 5 and fixed and stained for rods on day 6. Between 10–13% of neurons expressing cofilinR21Q-mRFP form mRFP-detectable rods in response to Aβd/t, about the same percentage of neurons with rods as we identified by cofilin immunolabelling of Aβd/t-treated mRFP expressing control cells (15%) (Figure 5A). A significant increase in rod number index was also observed for Aβd/t treated neurons, about 1.8 fold over their corresponding non-Aβd/t treated but adenoviral-infected controls (Figure 5B).

In contrast to AMPA treatment and ATP depletion, we found that only 48×4% (std. deviation; 147 neurons from 4 experiments) of the immunolabeled rods in neurons treated with Aβd/t contained cofilinR21Q-mRFP (Figure 4). The reasons for reduced incorporation of cofilinR21Q-mRFP into the Aβ-induced compared to AMPA-induced rods are not obvious but three possibilities come to mind, each of which has some supporting evidence. First, cofilinR21Q-mRFP expression levels in some processes might be below the threshold needed to clearly observe rods. We observe for any single neuron non equal distribution of cofilinR21Q-mRFP between neurites. Since the percent rod index and number rod index of cells expressing cofilinR21Q-mRFP decline as a function of decreased cofilinR21Q-mRFP expression (Figure 5), neurites containing low levels of the cofilinR21Q-mRFP may have rods in which the reporter is below the detection threshold. Second, slowly forming rods (such as those induced by Aβd/t) are more likely to reach an equilibrium binding that strongly favors the pool of endogenous ADF/cofilin which has higher affinity for F-actin than the cofilinR21Q-mRFP (Figure S3 in File S1). Third, recruitment of cofilin-mRFP (wt or mutant) could be enhanced by rod-associated ancillary proteins. Results from *in vitro* studies indicate that only actin and an ADF or cofilin are required to form the core rod structure [13]. However, rods formed rapidly in response to ATP depletion associate with other ancillary proteins that could bind preferentially to cofilinR21Q-mRFP and thus increase mRFP incorporation. Two such proteins, 14-3-3ζ [13] and a protein with a phosphorylated microtubule associated protein (MAP) epitope [41] are known to associate with rods induced by ATP depletion.

### Induced rods are dynamic and reverse upon stress removal

**Figure 6:**
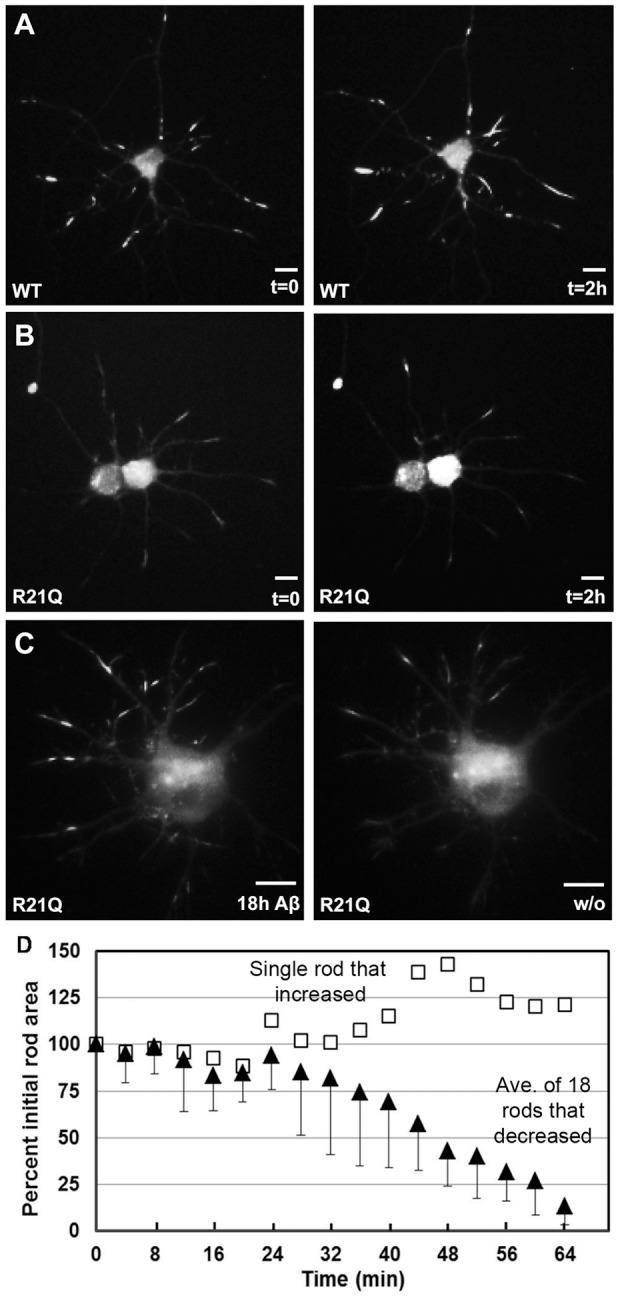
Visualization of rod formation and disappearance in neurons. Panels from time lapse imaging of neurons expressing cofilin-wt-mRFP (A) or cofilinR21Q-mRFP (B, C). In (A), neurons expressing cofilin-wt-mRFP had spontaneous rods present before imaging and acquired more rods due to photostress during the 2 h of imaging at 30 s intervals. Under identical imaging conditions, neurons expressing cofilinR21Q-mRFP had few spontaneous rods before imaging and did not form new rods during imaging (B). When cofilinR21Q-mRFP expressing neurons were treated with Aβd/t, formation of induced rods could be followed (C) that disappeared following removal of the Aβd/t (image of washout (w/o) is at 60 min). Scale bars  = 10 μm. (D) Disappearance of cofilinR21Q-mRFP rods induced by 18 h Aβd/t-treatment in images taken at 4 min intervals after Aβd/t washout (filled triangles). Total area of 18 rods in three separate neurons is plotted vs time after washout as a percent of the area at washout. The half-life of rods following Aβd/t removal is ∼ 47 min. One rod, in a separate neurite of a neuron that contained reversible rods in other neurites, was found to increase in size (open squares) and is presumed to be a spontaneous rod.

We previously showed that rods disappeared in neurons fixed after 24 h of recovery [8], [12]. To demonstrate that cofilinR21Q-mRFP can be used to study rod dynamics in real time, we performed several live cell imaging studies that also highlight its various potential applications. In the first series of experiments we used cofilinR21Q-mRFP to study formation and reversibility of Aβd/t-induced rods. Neurons were infected on day 3, treated with Aβd/t on day 4 and imaged on day 5. Because these studies were performed on a different microscope (Nikon TIRF) with different lasers from the ones used previously, we included neurons expressing cofilin-wt-mRFP for direct comparisons under identical imaging conditions. As expected, neurons expressing cofilin-wt-mRFP formed spontaneous rods and rod numbers increased during 2 h of imaging (Figure 6A). Neurons expressing cofilinR21Q-mRFP (at expression levels comparable to cofilin-wt-mRFP) have only an occasional rod, similar in number to immunolabeled uninfected or control infected (mRFP only) cultures. No additional rods formed during 2 h of imaging (Figure 6B). When neurons expressing cofilinR21Q-mRFP were treated with Aβd/t, rod formation occurred over several hours of imaging; when Aβd/t was removed by washing and medium replacement, most induced rods shrunk or disappeared during the next 60 min of imaging (Figure 6C). We calculated an average half-life of ∼47 min for rods after removal of Aβd/t (Figure 6D). Interestingly, one rod did not shrink and even enlarged somewhat during the 1 h observation period when all other rods in the same neuron (but different neurites) shrunk (Figure 6D), suggesting that it is a spontaneous and not an Aβd/t-induced rod. The rapid rate of rod reversal for induced rods suggests that their persistence requires continuous production of intracellular signals generated by externally applied Aβd/t. These studies affirm the applicability of cofilinR21Q-mRFP for studying the kinetics of induced rod formation and reversal.

**Figure 7:**
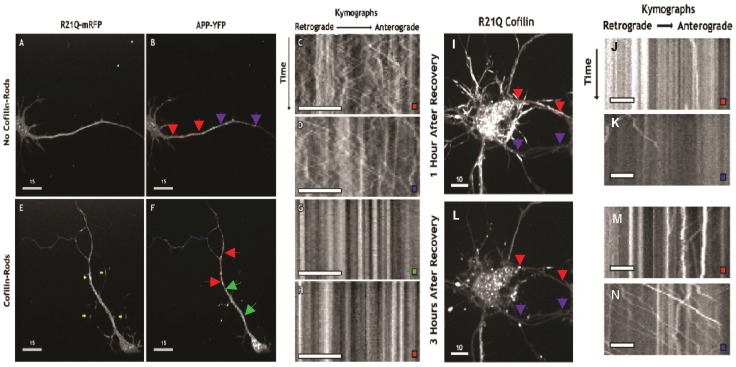
Live-imaging of AMPA-induced cofilinR21Q-mRFP labeled rods and their effect on vesicular transport before and after reversal. CofilinR21Q-mRFP and amyloid precursor protein (APP)-YFP dual infected neuronal cultures were treated with 25 μM AMPA for 45 min before imaging. Neurons expressing both fluorescent protein chimeras, which formed or did not form rods, were imaged once in the red channel to locate rods and then continuously for 12 sec (50 frames) in the YFP channel. In the AMPA-treated neuron that did not form rods (A), APP-YFP-containing vesicles (B) remain dynamic, as indicated by the retrograde (leftward) and anterograde (rightward) movements of the labeled vesicles in the kymographs (C, D) obtained from the regions of the neurite with red and purple line-scans (colored arrows demarcate the ends of the line scans) corresponding to the colored box on the kymographs. In the AMPA-treated neuron that formed rods (E, yellow arrows), APP-YFP-containing vesicles (F) stopped moving, as indicated by the lack of retrograde (leftward) and anterograde (rightward) movements of the labeled vesicles in the kymographs (G, H) obtained from the regions of the neurite with green and red line-scans (F) corresponding to the colored box on the kymographs. (Scale bars on images A, B, E, F are 15 μm and on kymographs C, D, G, H are 8 μm). In another set of experiments cofilinR21Q-mRFP and APP-YFP expressing neurons were treated 30 min with 25 μM AMPA to induce rods and then washed once with fresh medium and treated with 10 μM DNQX to prevent further AMPA-receptor activity. Neurons with rods were identified in the red channel. One hour after washout rods were still present in many processes (I) and APP-YFP containing vesicles were generally not dynamic, although a few exceptions were observed in kymographs taken along two different line scans (J, K). By three hours after washout (L), most rods have disappeared and APP-YFP vesicles are more dynamic (M, N). Line scans in I and L are not identical due to slight morphological changes that occur during recovery. (Scale bars on I, L = 10 μm and on kymographs J, K, M, N = 5 μm).

Cofilin-actin rods are thought to compromise synaptic function at least in part through their ability to block vesicular transport [11], [16]. However, this blockage has never been studied in live neurons in which rods can be reversed. The amyloid precursor protein (APP) is trafficked anterogradely from the trans-Golgi network to the plasma membrane and retrogradely within recycling endosomes for lysosomal degradation [42], [43]. Therefore, in a second set of experiments, we wanted to determine whether rod formation was associated with changes in motility of APP-YFP-containing vesicles. Rat hippocampal neurons overexpressing both cofilinR21Q-mRFP, to image rods, and APP-YFP, which is used as a membrane marker for vesicles, were treated with AMPA for 45 min to induce rods before live-cell imaging. Since not all neurons formed rods, we could compare the dynamics of APP-YFP-containing vesicle trafficking in a neuron with and a neuron without rods. In a neuron that did not form cofilin-actin rods, APP-YFP-containing vesicles were highly dynamic in nearly all neurite-processes observed, including presumptive dendrites and axons (Figure 7A–D). In the neuron that formed rods, a global loss of trafficking of APP-YFP-containing vesicles was observed (Figure 7E–H). The formation of rods in any one particular neurite was typically associated with the loss of trafficking throughout the entire neuron, even in neurites that did not contain an mRFP labeled rod (Figure 7E–H).

To determine whether the reversal of AMPA-induced rods was also associated with the resumption of trafficking of APP-YFP-containing vesicles, we imaged rods and APP-YFP in neurons over several hours after treating with DNQX, a drug that blocks AMPA-induced rods [14]. The reversal of rods 3 h after treating with DNQX was associated with the resumption of trafficking of APP-YFP-containing vesicles, although to different degrees within individual neurites (Figure 7I–N). Conversely, the presence of rods within the same neurites only 1 h after treating with DNQX was associated with the persistent disruption of APP-YFP-containing vesicle transport (Figure 7M). However, we did not see APP-YFP accumulation at either end of rods, which suggests either that the formation of rods has a more global effect within the neuron or that non rod-related processes, also essential for transport, are being compromised.

### Conclusions

CofilinR21Q-mRFP is a genetically encoded rod reporter that incorporates into cofilin actin rods within many different cell types and under different stress conditions without increasing spontaneous rod formation due to its own overexpression. Although it may not label all rods in all cases, especially slowly forming rods induced by Aβd/t, its expression does permit the study of rod dynamics and reversibility in response to addition and removal of stress-inducing agents. Reversal of rods has been understudied, and constitutes a potential novel approach for therapeutic intervention in a number of neurodegenerative disorders for which rods may be a common pathology resulting in synapse dysfunction. Future work will elucidate signaling pathways that initiate and/or maintain rod formation. Combined with other live-cell reporters, such as with genetically encoded sensors for Ca^2+^ [44] or sites of reactive oxygen production [45], cofilinR21Q-mRFP will be useful in determining spatial and temporal relationships between rod formation and synapse function.

## Supporting Information

File S1 **Combined file of all supporting figures whose legends are given below. Figure S1**. Levels of cofilin expression vary significantly depending on the promoter driving expression. N2a mouse neuroblastoma cells were infected with adenoviruses in which expression of cofilin-RFP was controlled by CMV, MCP, or NSE promoters (see Methods). At 72 h after infection, the amount of expressed cofilin-mRFP was quantified from Western blots and normalized to endogenous cofilin. Other cell types were used which also showed strong expression from CMV and moderate expression from MCP (SAOS2: CMV 4.5±0.7 fold; MCP 0.6±0.3 fold), but the NSE promoter was much less active in non-neuronal cells, so only results from the N2a cells are shown here. **Figure S2.** Photostress increases formation of spontaneous rods in neurons expressing cofilin wt-mRFP but not in neurons expressing cofilinR21Q-mRFP. Hippocampal neurons were infected with adenoviruses expressing cofilin wt-mRFP or cofilinR21Q-mRFP, driven by CMV, MCP and NSE. Three days post infection, cells were photostressed by 2 h of imaging at 30 second intervals. Over the 2 h session, neurons expressing cofilin wt-mRFP generated many new rods whose abundance was proportional to the relative levels of cofilin wt-mRFP expressed. No new rods were observed in any of the cofilinR21Q-mRFP expressing neurons, regardless of the promoter driving expression, and thus were not included on the graph. **Figure S3.** The affinity of cofilinR21Q for F-actin is decreased substantially below that of wt or cofilinR22Q as measured by F-actin sedimentation. (A) Various concentrations of cofilin (0, 2.5, 5, 10, 20 µM) were incubated with 5 µM F-actin at room temperature and after 10 min the samples were centrifuged at 250,000×g for 30 minutes at 20°C. For each concentration of cofilin assayed aliquots of the sample before centrifugation (T), the supernatant after centrifugation (S), and the pellet after centrifugation (P) were treated with SDS-sample preparation buffer and subjected to electrophoresis on 15% isocratic polyacrylamide gels and stained with Coomassie Blue R. (B) Bands on the stained gels (TotalLab software) were used to quantify the relative amount of cofilin that co-sedimented with the pellet-fraction (P) at different cofilin concentrations compared to the maximum wild type cofilin co-sedimenting. CofilinR21Q co-sediments with F-actin ∼9-fold less well than cofilin wt, and cofilinK22Q is intermediate in binding affinity. In the absence of F-actin, centrifugation of wild type and mutant cofilin resulted in no pelleted material or band on the gel (data not shown).
(DOCX)
