# Supplementary material for: A Genetically Encoded Reporter for Real-Time Imaging of Cofilin-Actin Rods in Living Neurons
Source: PLoS One. 2013 Dec 31;8(12):e83609. doi: 10.1371/journal.pone.0083609 (PMC3877059; doi:10.1371/journal.pone.0083609)
Supplement: File S1 — Combined file of all supporting figures whose legends are given below. Figure S1. Levels of cofilin expression vary significantly depending on the promoter driving expression. N2a mouse neuroblastoma cells were infected with adenoviruses in which expression of cofilin-RFP was controlled by CMV, MCP, or NSE promoters (see Methods). At 72 h after infection, the amount of expressed cofilin-mRFP was quantified from Western blots and normalized to endogenous cofilin. Other cell types were used which also showed strong expression from CMV and moderate expression from MCP (SAOS2: CMV 4.5±0.7 fold; MCP 0.6±0.3 fold), but the NSE promoter was much less active in non-neuronal cells, so only results from the N2a cells are shown here. Figure S2. Photostress increases formation of spontaneous rods in neurons expressing cofilin wt-mRFP but not in neurons expressing cofilinR21Q-mRFP. Hippocampal neurons were infected with adenoviruses expressing cofilin wt-mRFP or cofilinR21Q-mRFP, driven by CMV, MCP and NSE. Three days post infection, cells were photostressed by 2 h of imaging at 30 second intervals. Over the 2 h session, neurons expressing cofilin wt-mRFP generated many new rods whose abundance was proportional to the relative levels of cofilin wt-mRFP expressed. No new rods were observed in any of the cofilinR21Q-mRFP expressing neurons, regardless of the promoter driving expression, and thus were not included on the graph. Figure S3. The affinity of cofilinR21Q for F-actin is decreased substantially below that of wt or cofilinR22Q as measured by F-actin sedimentation. (A) Various concentrations of cofilin (0, 2.5, 5, 10, 20 µM) were incubated with 5 µM F-actin at room temperature and after 10 min the samples were centrifuged at 250,000×g for 30 minutes at 20°C. For each concentration of cofilin assayed aliquots of the sample before centrifugation (T), the supernatant after centrifugation (S), and the pellet after centrifugation (P) were treated with SDS-sample preparation buff [file pone.0083609.s001.docx]

**Supporting Information**

**A Genetically Encoded Reporter for Real-Time Imaging of Cofilin-Actin Rods in Living Neurons**

Jianjie Mi, Alisa E. Shaw, Chi W. Pak, Keifer Walsh, Laurie S. Minamide, Barbara W. Bernstein, Thomas B. Kuhn, and James R. Bamburg

Figure S1

Figure S2.

Figure S3
